# Which Functional Tests and Self-Reported Questionnaires Can Help Clinicians Make Valid Return to Sport Decisions in Patients With Chronic Ankle Instability? A Narrative Review and Expert Opinion

**DOI:** 10.3389/fspor.2022.902886

**Published:** 2022-05-26

**Authors:** Brice Picot, Alexandre Hardy, Romain Terrier, Bruno Tassignon, Ronny Lopes, François Fourchet

**Affiliations:** ^1^French Handball Federation, Creteil, France; ^2^French Society of Sports Physical Therapist (SFMKS Lab), Pierrefitte-sur-Seine, France; ^3^Inter-University Laboratory of Human Movement Biology (LIBM), Savoie Mont-Blanc University, Chambéry, France; ^4^Clinique du Sport Paris, Paris, France; ^5^SARL Whergo, Savoie Technolac (BP 80218), La Motte-Servolex, France; ^6^Human Physiology and Sports Physiotherapy Research Group, Faculty of Physical Education and Physiotherapy, Vrije Universiteit Brussel, Brussels, Belgium; ^7^Santé Atlantique, Pied Cheville Nantes Atlantique, Nantes, France; ^8^Motion Analysis Lab, Physiotherapy Department, La Tour Hospital, Swiss Olympic Medical Center, Meyrin, Switzerland

**Keywords:** return to sport (RTS), ankle, self-reported function, psychological readiness, instability, review (article), functional performance testing

## Abstract

Lateral ankle sprain is the most common injury in sports, with up to 40% of patients developing chronic ankle instability (CAI). One possible cause underlying this high rate of recurrence or feeling of giving way may be a premature return to sport (RTS). Indeed, except for time-based parameters, there are no specific criteria to guide clinicians in their RTS decisions in patients with CAI. A recent international consensus highlighted the relevance and importance of including patient-reported ankle function questionnaires combined with functional tests targeting ankle impairments in this population. Thus, the aim of this narrative review and expert opinion was to identify the most relevant functional performance tests and self-reported questionnaires to help clinicians in their RTS decision-making process following recurrent ankle sprains or surgical ankle stabilization. The PubMed (MEDLINE), PEDro, Cochrane Library and ScienceDirect databases were searched to identify published articles. Results showed that the single leg stance test on firm surfaces, the modified version of the star excursion balance test, the side hop test and the figure-of-8 test appeared to be the most relevant functional performance tests to target ankle impairments in patients with CAI. A combination of the Foot and Ankle Ability Measure (FAAM) and the Ankle Ligament Reconstruction-Return to Sport after Injury (ALR-RSI) questionnaires were the most relevant self-reported questionnaires to assess patient function in the context of CAI. Although these functional tests and questionnaires provide a solid foundation for clinicians to validate their RTS decisions in patient with CAI, objective scientific criteria with cut-off scores are still lacking. In addition to the proposed test cluster, an analysis of the context, in particular characteristics related to sports (e.g., fatigue, cognitive constraints), to obtain more information about the patient's risk of recurrent injury could be of added value when making a RTS decision in patients with CAI. In order to evaluate the strength of evertors under ecological conditions, it would also be interesting to assess the ability to control weight-bearing ankle inversion in a unipodal stance. Further studies are needed to assess the relevance of this proposed test cluster in RTS decision-making following lateral ankle sprain injury and CAI.

## Introduction

Lateral ankle sprain (LAS) is one of the most common musculoskeletal injuries in the general population and is the most frequently reported by athletes (Gribble et al., 2014; Hertel and Corbett, 2019). According to the International Ankle Consortium, a LAS is defined as “*an acute traumatic injury to the lateral ligament complex of the ankle joint as a result of excessive inversion of the rear foot or a combined adduction of the foot*” (Gribble et al., 2014). Data from emergency departments suggest an incidence rate of 2.1–3.2 acute LAS/1,000 person-years in the general population (Herzog et al., 2019). The incidence rates of ankle sprains are 5.5 times higher than those registered at emergency departments (Kemler et al., 2015) and probably about one patient out of two does not seek medical attention for this common injury (Gribble et al., 2016). Finally, it is worth noting that the ankle is the area with the highest number of misdiagnoses in emergency departments (Moonen et al., 2017) and that <10% of patients who consulted will undergo rehabilitation within 1 month of the injury (Martin et al., 2021).

The prevalence of LAS, associated with high rates of recurrence, persistent impairments, deterioration of functional ankle capacity and long-term sequelae are a real public health burden (Vuurberg et al., 2018) with an estimated total cost ranging from €360 to 1,100 per individual. This disparity in costs is due to variations in the healthcare system, population, and type and severity of injury (Vuurberg et al., 2018). This is reflected in the notion of chronic ankle instability (CAI): a condition characterized by frequent episodes of giving way, permanent symptoms such as pain, weakness or reduced ankle range of motion, decreased self-supporting function and recurrent ankle sprains that persist for more than a year after the initial injury. The updated model from Hertel and Corbett (2019) suggests that CAI is a multifaceted problem that affects several functional abilities and specific diagnostic criteria for CAI have been recommended by the International Ankle Consortium (Gribble et al., 2014). If medical treatment of CAI is unsuccessful, instability episodes should be controlled to avoid post-traumatic ankle osteoarthritis which can develop in 68–78% of patients with CAI (Harrington, 1979; Wikstrom et al., 2013). Surgical treatment should be considered in these cases to restore stability of the ankle because the longer instability remains untreated, the higher is the risk of osteoarthritis (Wang et al., 2020).

Copers are defined as “*individuals who had an ankle sprain, but did not go on to develop CAI”* (Wikstrom and Brown, 2014) so the goal of rehabilitation after an acute ankle sprain is for individuals to become copers instead of becoming patients with CAI. These authors have clearly defined these individuals based on three main characteristics, in particular (Gribble et al., 2014) an initial ankle sprain severe enough to warrant either the use of a protective device for at least 1 week and/or non-weight bearing for at least 3 days; (Hertel and Corbett, 2019) a return to at least moderate levels of weight-bearing physical activity for at least 12 months without recurrent injury, episodes of giving way and/or feelings of instability; and (Herzog et al., 2019) lack of self-reported disability.

Premature RTS in patients with CAI could play a role in the development of persistent ankle instability and the high prevalence of recurrent ankle injuries (Medina McKeon et al., 2014). Current RTS decision-making is further complicated by an absence of prospective studies evaluating RTS criteria following LAS (Tassignon et al., 2019; Wikstrom et al., 2020). A Delphi approach was recently used to establish a consensus opinion from a panel of international healthcare professionals specialized in the follow-up and RTS decision-making process for high level athletes (Smith et al., 2021). These experts reached a consensus on 16 items that could be included as RTS criteria in individuals following LAS. These items include sport-specific tasks, hopping, agility, jumping, pain severity during sport-specific activity and in the last 24 h, ankle strength/endurance and range of motion, dynamic postural control, proprioception, perceived ankle reassurance, perceived ankle instability and psychological readiness. The authors classified these parameters into five sections included in the **PAASS** acronym for: **P**ain, **A**nkle impairments, **A**thlete perception, **S**ensorimotor control and **S**port/functional performance. Scientifically proven and prospectively determined RTS criteria are also lacking in CAI populations. Although this study helps clinicians choose which items to assess, it does not specify which tools they should use to measure them. Furthermore, the authors do not propose measurement thresholds that indicate that the athlete is ready to RTS. Thus, in clinical practice RTS decisions are mainly based upon the experience, expertise and clinical reasoning of the clinician managing the patient with CAI. Nevertheless, authors advocate that including relevant questionnaires and functional tests to validate RTS decisions in CAI populations could markedly improve the quality of the RTS decisions in clinical practice. Indeed, patient reported outcome measures (PROM) have been shown to be useful in the management of several injuries (Fitzpatrick et al., 1998; Dawson et al., 2010; Black, 2013) including those affecting foot and ankle (Hunt and Hurwit, 2013; Jia et al., 2017; Anderson et al., 2018).

The aim of this narrative review and expert opinion was therefore to identify the most relevant functional tests and self-reported questionnaires following ankle sprains or surgical ankle stabilization. We aimed to identify the most appropriate tools to target sensorimotor impairments, athlete perception and functional performance and suggest relevant cut-off scores. This is a first step to help clinicians in their RTS decision-making process with patients with CAI.

## Materials and Methods

An exhaustive review of the literature published until January 1, 2022 was independently performed by three researchers (BP, RT, and FF) in the PubMed (MEDLINE), PEDro, Cochrane Library and ScienceDirect databases. This included clinical trials, consensus statements, systematic reviews and meta-analyses related to functional tests as well as self-reported questionnaires following ankle sprains or ankle surgical stabilization in the process of RTS. A primary search using the following keywords: (1) ankle injuries (Mesh term NOT “syndesmotic”) OR “ankle instability” (2) “return to sport” (Mesh Term) (3) “Functional Performance Test” OR “self-reported questionnaire” OR “psychological readiness” was performed. Key search terms were determined by our purpose and research question and confirmed by expert opinion of all of the investigators. A secondary search was performed through the references of included studies and relevant review articles identified from the primary search were also included. We mainly selected assessments that revealed significant differences between patients with CAI compared to copers or healthy individuals. When available, we chose the scores obtained by copers rather than healthy subjects as reference values.

As both static and dynamic postural deficits contribute to CAI, we selected the most reliable and clinically relevant functional tests to target these deficits. Moreover, based on the same criterion, agility and hopping tests were included if they could differentiate individuals with CAI from copers or healthy individuals. It is now well-established that CAI contributes to self-reported deficits and that psychological readiness is a key factor in the RTS process following lateral ankle sprains. We therefore only included questionnaires that monitor region-specific function (i.e., foot and ankle) or the psychological features in patients with CAI. The final decision to include measurement tools also depended on the reliability, relevance and the ability to use them in daily practice (no specific or expensive equipment).

Based on the above criteria, a consensus was reached by all co-authors on the final choice of tests and questionnaires identified in the literature as well as proposed cut-off scores to help practitioners to decide on the RTS according to the available literature. The reliability and clinical relevance of each item was also searched for to guide clinicians in the interpretation of their patients' results. The intraclass correlation coefficient (ICC), Standard Error of Measurement (SEM), Minimal Detectable Change (MDC) and the minimal clinically important differences (MCID) were therefore reported if available in patients with CAI.

## Results

Table 1 summarizes the reliability, clinical relevance and proposed cut-off points of all selected tests or questionnaires. Overall, these tests evaluate static and dynamic postural control, hopping and agility, self-reported function and psychological readiness in patients with CAI.

**Table 1:** Summary of main functional tests and patient reported outcome measures (PROM) as relevant return to sport criteria.

**Outcomes**	**Proposed cut off score**	**Reliability**		**MDC**	**MCID**
**Functional performance testing**					
*Single leg stance on firm surface* (Riemann et al., 1999; Docherty et al., 2005; Linens et al., 2014)	<3 errors	Interrater: ICC = 0.93 and SEM = 0.45		0.6 errors	NR
*Foot lift Test* (Docherty et al., 2005; Hiller et al., 2007; Linens et al., 2014; Ko et al., 2017; Cain et al., 2020)	<5 lifts	ICC = 0.73 (0.40–0.89) ICC = 0.97 and SEM = 1.3 error		3 errors	NR
*Star excursion balance test* (normalized to the leg length) (Butler et al., 2013; Linens et al., 2014; Stiffler et al., 2015, 2017; Powden et al., 2019; Picot et al., 2021; Udompanich et al., 2021)		ICC Inter rater:	ICC Intra rater:		NR
*Composite score (COMP)*	COMP > 90%	COMP = 0.91–0.93	COMP = 0.93–0.94	COMP = 6.7%	
*Anterior (ANT)*	ANT asymmetry <4.5% or 4 cm	ANT = 0.88 (0.83–0.96)	ANT = 0.88 (0.84–0.96)	ANT = 5.87%	
*Posteromedial (PM)*	PM > 91%	PM = 0.87 (0.8–1.0)	PM = 0.88 (0.85–0.94)	PM = 7.84%	
*Posterolateral (PL)*	PL > 91%	PL = 0.88 (0.73–1.0)	PL = 0.90 (0.68–0.94)	PL = 7.55%	
*Side hop test* (Caffrey et al., 2009)	<10 s	Test retest: ICC = 0.84, SEM = 2.10 s		5.82 s	NR
*Figure-of-8 hop test* (Caffrey et al., 2009)	<12 s	Test retest: ICC = 0.95, SEM = 1.66s		4.59 s	NR
**Patient reported outcome measure**					
*FAAM*_adl_ *andFAAM*_sport_ (Martin et al., 2005; Hoch et al., 2012; Hertel and Corbett, 2019)	95% for both score	Test retest: ICC= 0.89 (FAAM_adl_) ICC= 0.87 (FAAM_sport_)		FAAM_adl_=3.96 FAAM_sport_=7.9	FAAM_adl_: 8 FAAM_sport_: 9
*ALR-RSI* (Sigonney et al., 2020)	55/120	Test retest: ICC = 0.92 (0.86–0.96)		10 points	NR

*ICC, Intraclass correlation coefficient; SEM, Standard Error of Measurement; MDC, minimal detectable Change; MCID, minimal clinically important difference; FAAM, Foot and Ankle Ability Measure; ALR-RSI, Ankle ligament reconstruction-return to sport after injury*.

### Single Leg Stance Test on Firm Surface

**Figure 1:**
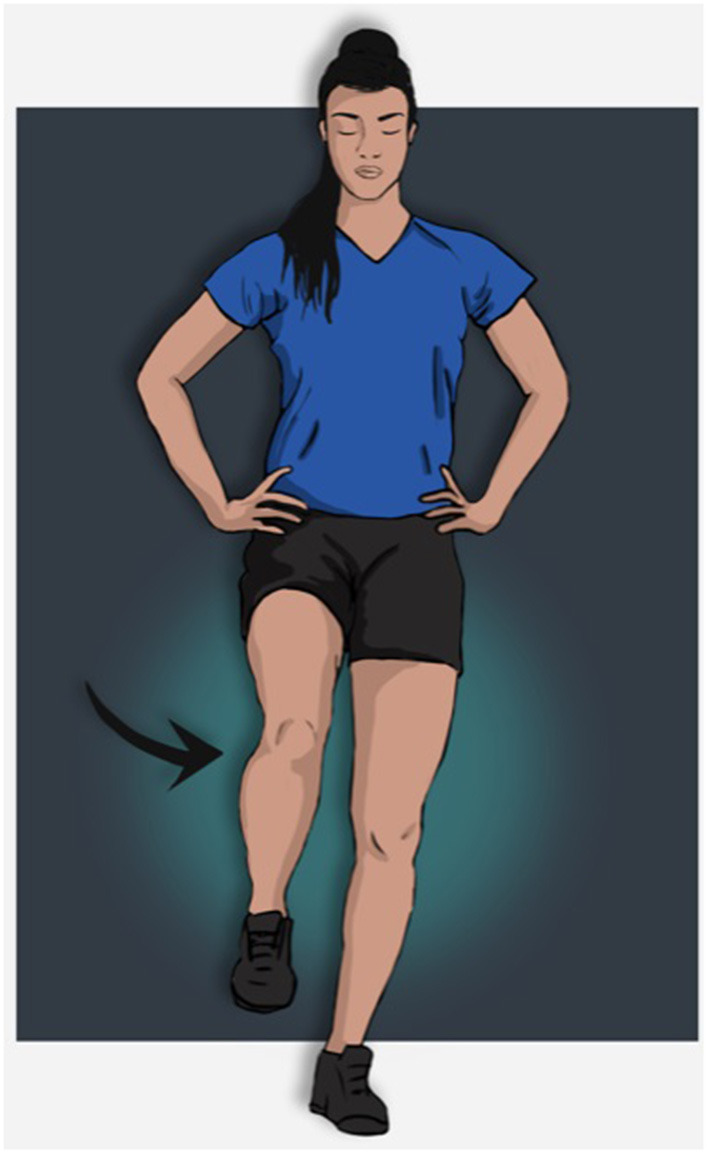
Single leg stance on firm surface or foot lift test of the left limb.

This test is derived from the Balance Error Scoring System (Riemann et al., 1999; Docherty et al., 2006; McKeon and Hertel, 2008; Bell et al., 2011) and is only performed on a firm surface. It is one of the most common tests described to assess static postural control (Gribble et al., 2012; Cain et al., 2020). Previous studies have found inter-rater reliability to be good with ICC values of 0.93 and a SEM of 0.45 (Riemann et al., 1999). Participants stand barefoot on the tested limb, look straight ahead and are then instructed to keep their eyes closed and their hands on their hips for 20s (Figure 1). The test must be performed with the weight-bearing leg at ~5° of knee flexion and with the hip and knee of the non-weight-bearing limb slightly flexed (Riemann et al., 1999). The examiner counts the number of balance errors that occur during the test (Table 2). Familiarization is allowed with several practice trials before performing the test. The total number of errors committed in the trial of each leg are used for analysis (Linens et al., 2014). The single leg stance (SLS) test on a firm surface is now considered to be a relevant static functional balance test to identify CAI individuals (Arnold et al., 2009). Although patients with CAI have significantly poorer static postural control on a firm surface (2.9 ± 2.1 vs. 1.6 ± 1.3 errors) compared to healthy individuals (Docherty et al., 2006), no specific cut-off score has been clearly established. Furthermore, Linens et al. proposed a 3-error cut-off score because CAI individuals have more balance errors than healthy participants (2.53 ± 2.37 vs. 1.29 ± 1.05) (Linens et al., 2014).

**Table 2:** Single leg stance test on firm surface derived from the Balance Error Scoring System (BESS).

**Balance errors assessed by the examiner**
Lifting hands off iliac crest
Opening eyes
Stepping, stumbling or falling
Moving hip into more than 30° of flexion or abduction
Lifting forefoot or heel
Remaining out of the test position more than 5 s

### Foot Lift Test

A very similar test called the foot lift test (FLT) was developed based on foot displacement (Hiller et al., 2007). The guidelines and the set-up are quite similar to the Single Leg Stance test (Figure 1), but another error is added every times a part of the foot was lifted during the test. A “part foot lift” is defined as any part of the foot, such as toes or metatarsal heads, lifting from the floor. If the contralateral foot touched the floor, one count was added and an extra count for each second it remained on the floor (Hiller et al., 2007; Ko et al., 2017). Familiarization is allowed with several practice trials before performing the test. The total number of errors committed in the trial of each leg are used for analysis. Test and re-test reliability among individuals suffering from CAI was found to be good (ICC_2,1_ = 0.73, 95% CI = 0.40–0.89) to excellent 0.97 (SEM = 1.3 error) by Hiller et al. (2007) and Ko et al. (2017). This test is slightly different from the Single Leg Stance on a firm surface because it lasts for 30 s rather than 20 s (Figure 1). Furthermore, it focuses on small movements of the foot, while the SLS targets the eyes, hips, and hands (Linens et al., 2014). In a recent meta-analysis Rosen et al. found a large and significant mean difference (*g* = −0.761, *p* = 0.02) in foot lift test results between healthy and CAI individuals in three studies and the foot-lift test was reported to be a good discriminatory test between these populations (Hiller et al., 2007; Linens et al., 2014; Ko et al., 2017; Rosen et al., 2019). Nevertheless, these results must be considered with caution as several cut-offs were found to have marked discrepancies (errors ranging from 5 to 9) (Hiller et al., 2007; Linens et al., 2014; Ko et al., 2017; Udompanich et al., 2021). Only one study (Cain et al., 2020) reported the MDC in adolescent athletes with CAI.

### Star Excursion Balance Test

**Figure 2:**
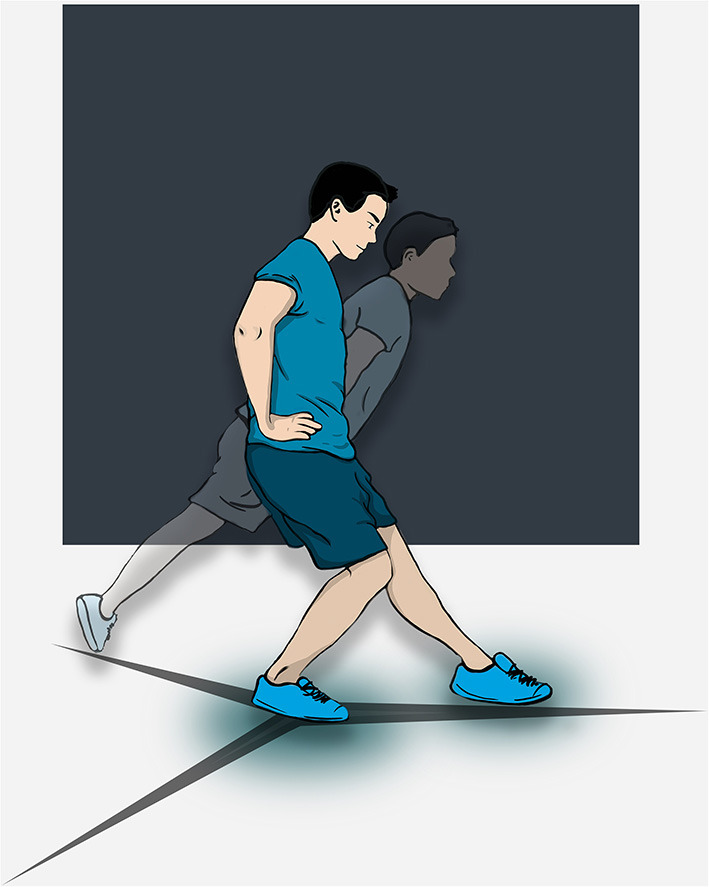
Star excursion balance test of the right limb.

The SEBT is a reliable functional test to evaluate dynamic postural control of the lower limb and distinguish CAI from copers and healthy individuals (Olmsted et al., 2002; Gribble et al., 2012). The median inter-rater reliability ICC values were 0.88 (0.83–0.96), 0.87 (0.80–1.0), and 0.88 (0.73–1.0) for the anterior (ANT), posteromedial (PM) and posterolateral (PL) directions, respectively. Although the systematic review by Gribble et al. (2012) provides recommendations for the most reliable procedure, various protocols have been described in the literature. Recent practical guidelines emphasize the need for consistent procedures (Picot et al., 2021). The most common and reliable method requires four practice trials in the ANT, PM, and PL directions followed by three recorded trials on each leg. Subjects stand barefoot on the tested limb, with the hands on the hips while they must reach the maximum distance with the opposite foot and return to the initial position without losing their balance (Figure 2). The trial is canceled if the subject lifts any part of the stance foot, removes his/her hands from the hips or transfers weight to the other limb. The distance is recorded (in cm) and evaluated in relation to the limb length (from the anterior and superior iliac spine to the medial malleolus). As recently discussed by Picot et al. (2021) everal foot positions have been also described during the test and could result in significant misinterpretation when comparing studies. The most commonly used procedure, especially in large cohorts, is described with the most distal aspect of the big toe at the intersection of the three directions. The average of the 3 trials is used to analyze each outcome measure. Normalized reach distances (i.e., percentage of limb length) for the anterior (ANT), posteromedial (PM) and posterolateral (PL) directions are calculated from the following equation.


$$\begin{array}{l} given\;score\;\left(\%\right)\\ =\frac{mean\;of\;the\;three\;trials\;in\;given\;direction\;\left(cm\right)\;}{tested\;limb\;length\;\left(cm\right)\;}\;\times 100 \end{array}$$

The mean of each direction is then used to calculate the composite score (COMP) using the following equation:


$$\begin{array}{l} normComposite\;score\;\left(\%\right)\\ =\frac{normANT\;\left(\%\right)+normPM\left(\%\right)+normPL\left(\%\right)}{3} \end{array}$$

Doherty et al. (2015, 2016) reported significant differences in all three directions in patients with CAI compared to healthy individuals 6 months and 1 year after LAS, with the largest observed effect size in the PL direction. However, since the performances in this test appear to be sport-dependent (Stiffler et al., 2015), various cut-off scores are reported in the literature. The recent meta-analysis by Rosen et al. (2019) shows that the PM direction is the most relevant outcome to distinguish CAI from healthy individuals. In this specific direction, a cut-off score of 91% was proposed (Linens et al., 2014; Udompanich et al., 2021) with a 2.20–2.55 positive likelihood ratio and a 0.36–0.5 negative likelihood ratio. Moreover, the asymmetry of the reach distances in the ANT direction appear to be a key factor for lower limb injuries because an absolute asymmetry ≥4 cm was associated with a 2.5 times increased risk and a normalized asymmetry of >4.5% identified athletes at an increased risk with a 82% accuracy in a large cohort (Plisky et al., 2006; Stiffler et al., 2015, 2017). Regarding the composite score, results from Plisky et al. (2006) revealed females who displayed a normalized composite score below 94% were 6.5 times more at risk of sustaining lower limb injury during the season. For males, the risk was three times higher among players who did not reached 94% of the lower limb length (Plisky et al., 2006). A cut off score of 90% seems more reasonable based on the results of Butler et al. (2013) who showed that a college soccer player who scored below 89.6% had 3.5 times the risk of injury.

### Side Hop Test

**Figure 3:**
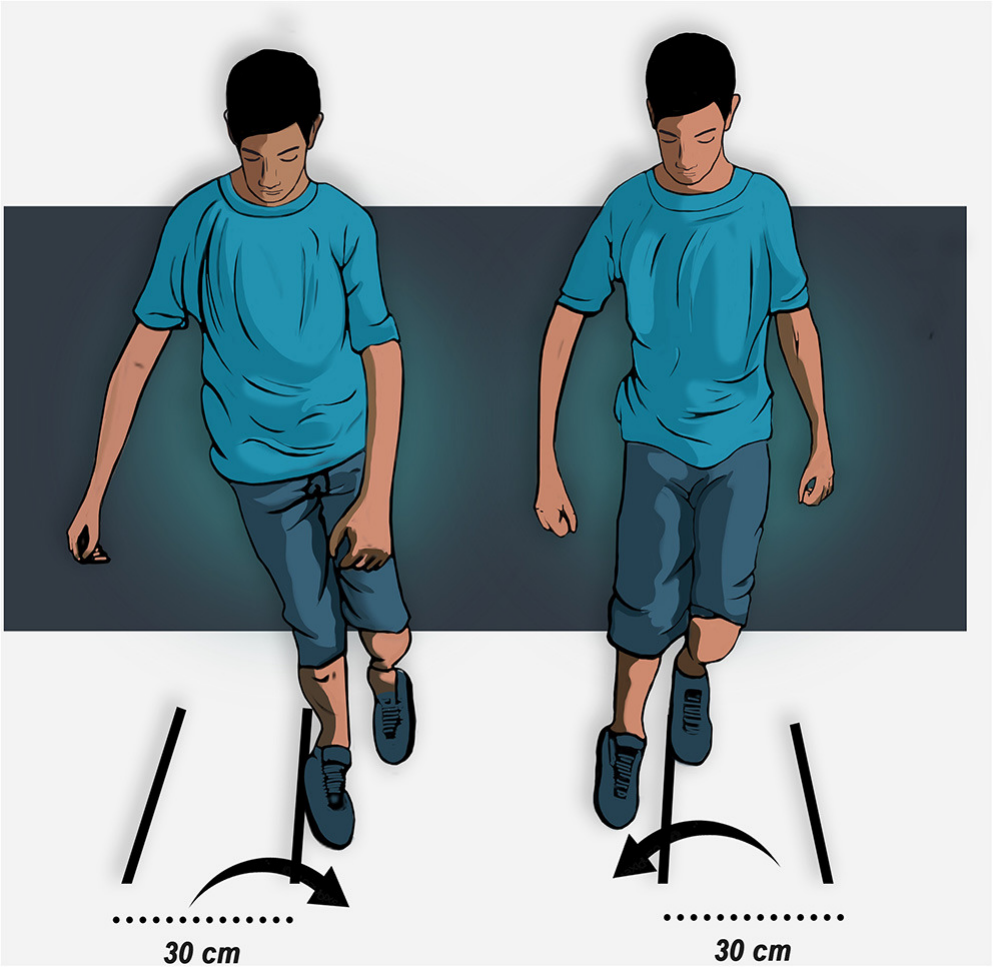
Side hop test of the right limb.

The performance of the side hop test (SHT) by individuals suffering from CAI is usually poorer in their injured limb than in their uninjured limb as well as than in copers and healthy people (Docherty et al., 2005). The SHT requires a significant amount of peroneus longus activation, which may be deficient in patients with CAI (Rosen et al., 2019). The patient is instructed to hop 10 times laterally and medially as quickly as possible over a 30 cm distance per trial for a total of 20 jumps (Figure 3) (Docherty et al., 2005; Caffrey et al., 2009; Sharma et al., 2011; Udompanich et al., 2021). At least two trials of this test are advised with a practice trial before performing the test. The test is performed with a 1-min rest period between trials. The fastest time to completion is selected as the final test score. Another important subjective feature that clinicians can consider besides the completed time is perceived ankle instability during the side hop test.

Because this hop test places higher demands on the ankle joint on the frontal plane, it identifies more functional stability deficits among individuals with CAI. Rehabilitation focused on balance and strength improves the performance of the injured limb in patients (Docherty et al., 2005; Caffrey et al., 2009; Wikstrom et al., 2009; Sharma et al., 2011; Linens et al., 2014; Cain et al., 2017, 2020; Wright et al., 2017; Madsen et al., 2018; Udompanich et al., 2021). The study by Udompanich et al. (2021) showed that patients with CAI with better balance performed better on the SHT than individuals with poorer balance. The meta-analysis from Rosen et al. (2019) confirmed the relevance of this functional test following ankle sprain with a cut-off score of 10 s to distinguish healthy participants from CAI individuals (Caffrey et al., 2009). The SHT includes important features of sport movements, such as cutting and landing. This functional test can be used both at the beginning of rehabilitation and to make a decision to RTS. Furthermore, the SHT could be more important in the RTS decision-making process for patients performing sports with more cutting and landing maneuver's (e.g., soccer, volleyball, dance).

### Figure-of-8 Hop Test

**Figure 4:**
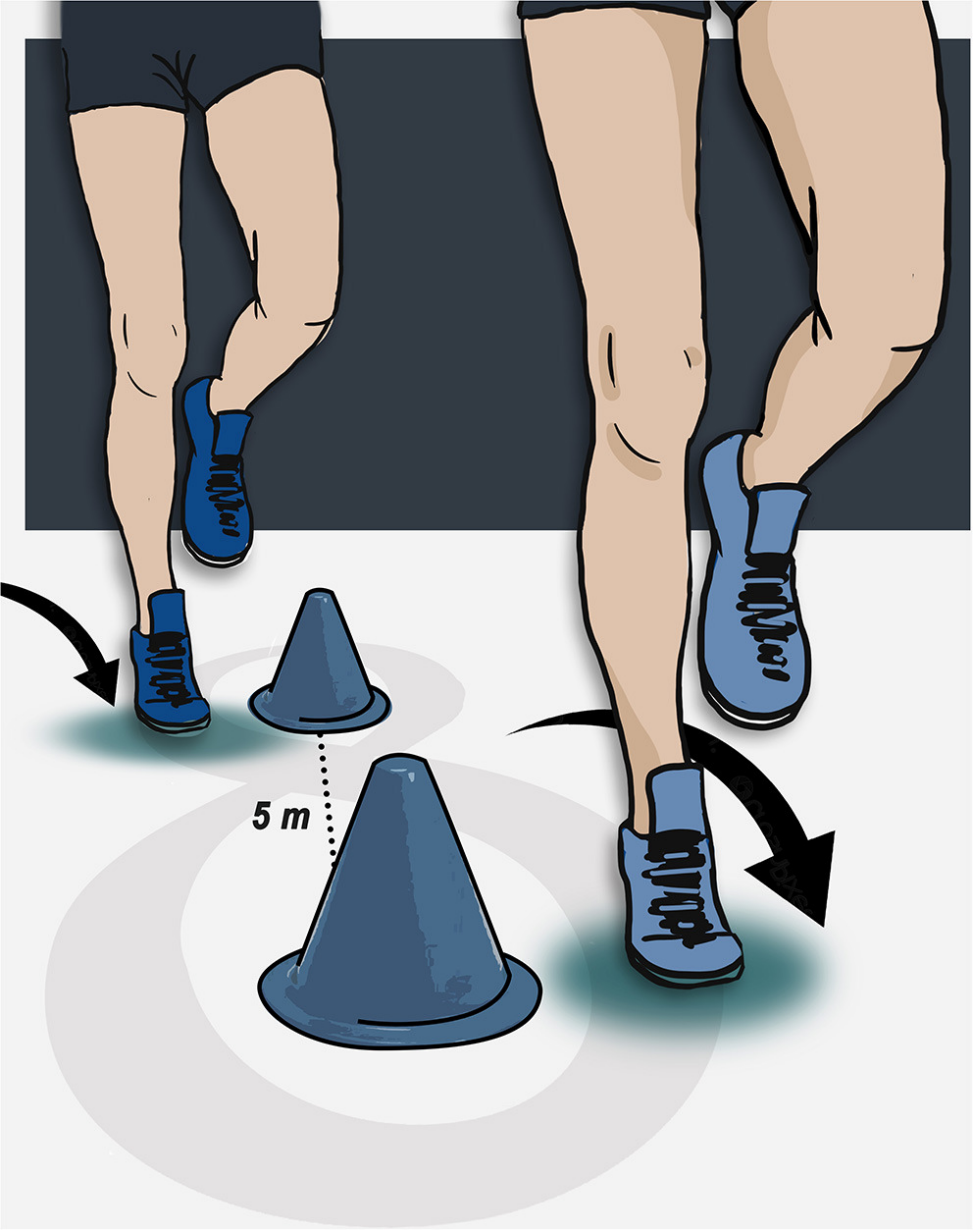
Figure-of-8 test of the right limb.

This functional performance test cannot always distinguish individuals suffering from CAI from copers or healthy people (Docherty et al., 2005; Caffrey et al., 2009; Wikstrom et al., 2009; Sharma et al., 2011; Linens et al., 2016; Cain et al., 2017, 2020; Wright et al., 2017; Madsen et al., 2018; Udompanich et al., 2021). Nevertheless, rehabilitation studies show improvement in this test among patients with CAI (Linens et al., 2016; Wright et al., 2017; Cain et al., 2020). Like the SHT, patients more often perceive subjective ankle instability than copers or healthy controls (Wikstrom et al., 2009; Wright et al., 2017). The patient is instructed to hop on one limb in a **Figure 8** pattern as fast as possible between two cones 5 meters apart (Figure 4). The patient has to perform two consecutive laps (for a total distance of 20 m) to complete this test (Caffrey et al., 2009; Sharma et al., 2011; Rosen et al., 2019). Participants are allowed to practice trials before performing the test. Similar to the SHT, the figure-of-8 hop test can also be used at the beginning of rehabilitation to measure potential deficits as well as being included as a functional test when making a RTS decision. To our knowledge, no previous threshold has been reported, but the control subjects in the 2005 study by Caffrey et al. yielded a meantime of 11.0 ± 0.4 s. As the aim of the review is to move forward and help clinicians in their daily practice, we suggest targeting this value which has a very narrow dispersion. Indeed, very few control subjects scored above 12 s. We encourage clinicians to use this follow-up test throughout rehabilitation since in all studies that have used the SHT, the CAI groups had scores higher than >13 s, but at the end of the rehabilitation process, they all approach the proposed threshold <12 s or do better.

### Foot and Ankle Ability Measure

**Figure 5:**
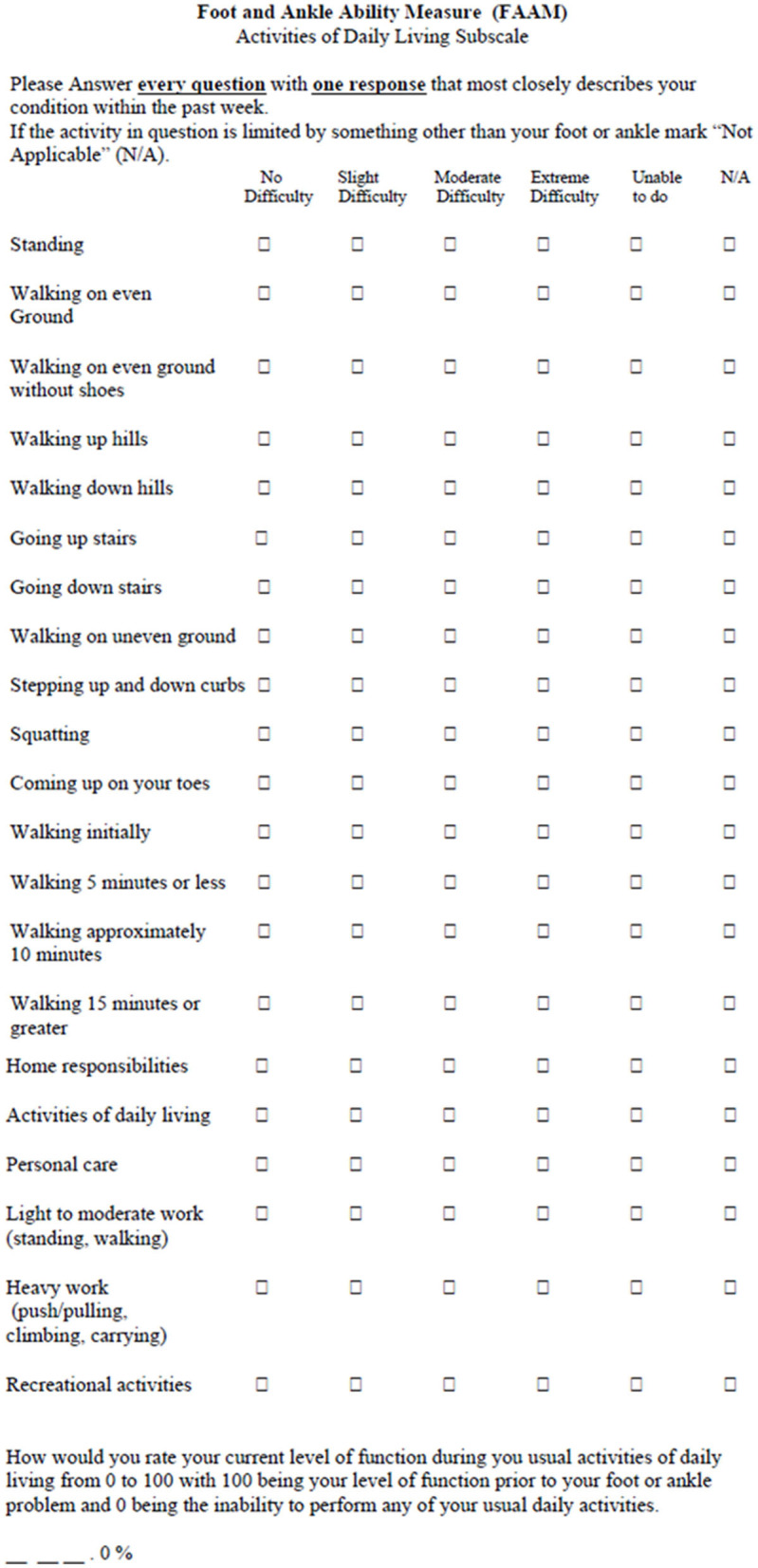
Foot and ankle ability measure, activities of daily living subscale (FAAM_adl_).

**Figure 6:**
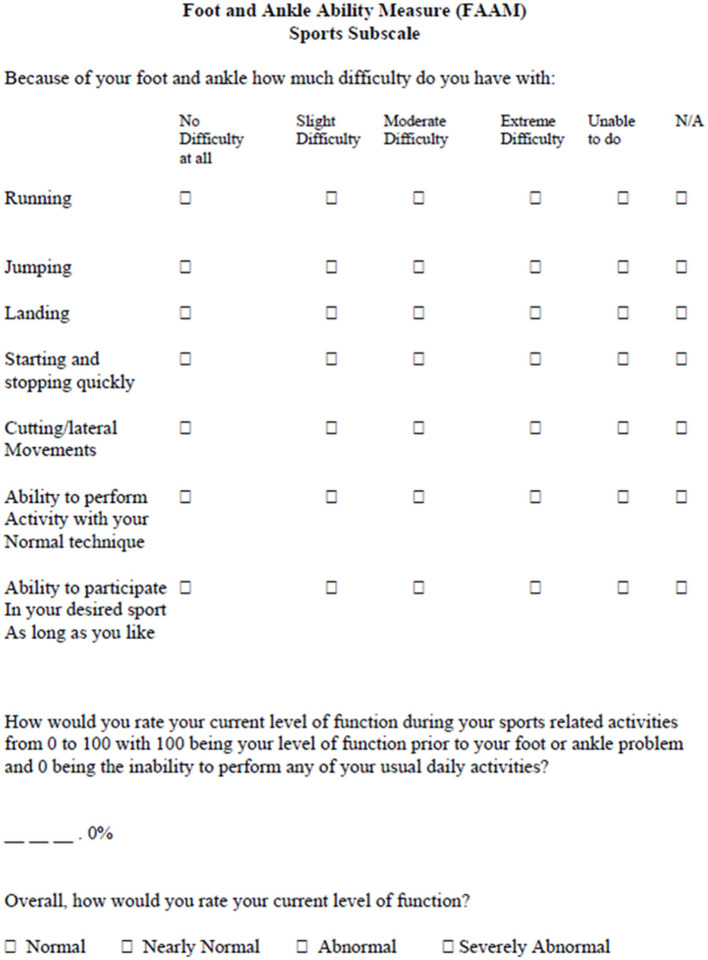
Foot and ankle ability measure, sports subscale (FAAM_sport_).

The use of functional evaluation scores is recommended in the management of patients with ankle instability (Martin et al., 2013, 2021). More than 130 scores have been described to evaluate the foot and ankle (Guillo et al., 2013; Hunt and Hurwit, 2013; Zwiers et al., 2018) with very different rates of reliability and validity. Certain scores are extremely generic and evaluate the entire foot/ankle complex (Kitaoka et al., 1994) while others are more specific and have been validated for a specific joint, or for chronic ankle instability (Donahue et al., 2011). At present there is no consensus on the use of these scores (Zwiers et al., 2018). The International Ankle Consortium recommends 2 scores to define the precise criteria for chronic ankle instability (Gribble et al., 2014), the Foot and Ankle Outcome Score (FAOS) (Roos et al., 2001) and the Foot and Ankle Ability Measure (FAAM) (Martin et al., 2005). The FAOS is a strict adaptation of the Knee Injury and Osteoarthritis Outcome Score (KOOS) proposed by the same author and composed of 42 identical items. The meta-analysis by Houston on the different questionnaires evaluating patient-reported function (Houston et al., 2015) recommends using the FAAM because of its short format, its high level of validation and because it is also the only questionnaire with the MDIC described by the literature. The FAAM is a self-assessment tool for individuals with musculoskeletal difficulties of the ankle and foot. First described by Martin et al. (2005) it is composed of 29 items divided into two subscales; the FAAM 21-items Activities of Daily Living subscale (Figure 5) and 8 item sports subscale (Figure 6). It was validated in 2008 for chronic ankle instability (Carcia et al., 2008) and translated into several languages (Borloz et al., 2011; Cervera-Garvi et al., 2017) with a recently validated computerized version of this questionnaire (Uimonen et al., 2021). The FAAM asks patients to evaluate their difficulty in performing day to day activities or sports because of their injured ankle. The International Ankle Consortium first proposed, using threshold values of 90% for the FAAM_adl_ and 80% for the FAAM_sport_, to identify patients with chronic ankle instability (Gribble et al., 2014). The range of the effects in healthy vs. unstable ankles was from 0.96 to 3.29, which indicates that this test correctly identifies functional deficits of the ankle in those with chronic instability (Houston et al., 2015). Moreover, a strong effect (*g* range: 0.75–1.73) was identified between patients with CAI and copers, confirming the important loss of function in daily life.

Since several studies have shown that healthy subjects report scores of 100% for the two subcategories. Hertel and Corbett (2019) argued therefore, in the updated model of CAI, that individuals should be above a threshold of 95% in both domains to be considered copers. On the other hand, 6 months after a lateral ankle sprain, the mean scores were 95.8 and 87.1 for FAAM_adl_ and FAAM_sport_ respectively (Doherty et al., 2015), while they were 95.7 and 85.5 at 1 year for patients with CAI compared to 98 and 90.6 in copers, respectively (Doherty et al., 2016). The MDC, initially calculated in a heterogenous population (Martin et al., 2005), was 5.7 and 12.3 points for the FAAM_adl_ and FAAM_sport_, respectively, but a more recent study among individuals with CAI adjusted the MDC of 3.96 and 7.90 points for FAAM_adl_ and FAAM_sport_, respectively (Hoch et al., 2012).

### Ankle Ligament Reconstruction-Return to Sport After Injury

**Figure 7:**
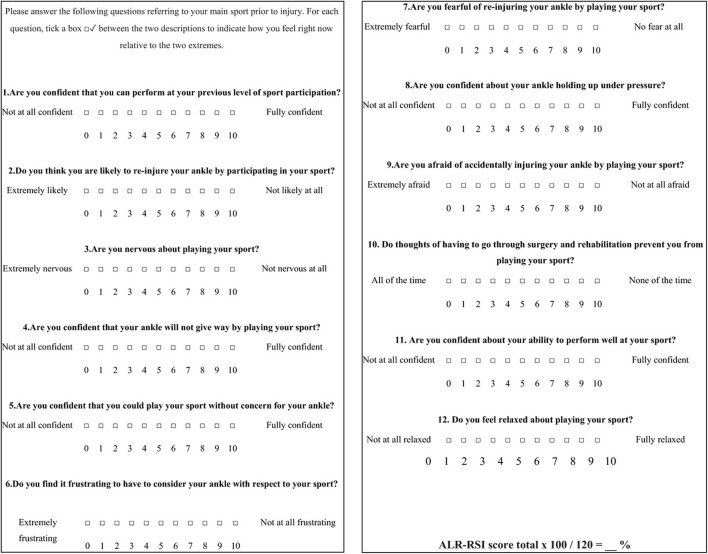
Ankle ligament reconstruction-return to sport after injury (ALR-RSI).

The key role of psychological factors for a successful RTS after sport's injuries has been confirmed (Podlog et al., 2014), especially following Anterior Cruciate Ligament reconstruction (ACL-R) (Ardern et al., 2014). Indeed, the RTS was mainly dependent upon the fear of re-injury, a lack of motivation, self-esteem, and confidence in the reconstructed knee as well as on the locus of control and self-efficacy (Bauer et al., 2014; Tassignon et al., 2019). Thus, recent reviews (Clanton et al., 2012; Tassignon et al., 2019) suggest that questionnaires to measure psychological readiness among patients should be used in CAI for example the Injury-Psychological Readiness to Return to Sport (I-PRRS), Trait Sport-Confidence Inventory (TSCI) or State Sport-Confidence Inventory (SSCI) (Vealey, 1986; Glazer, 2009). Unfortunately, none of these tests are specific for foot or ankle injuries or chronic instability. The Ankle Ligament Reconstruction-Return to Sport after Injury (ALR-RSI) is a psychological score to assess return to sport readiness after surgical reconstruction of the ankle or conservative treatment of ankle sprain (Sigonney et al., 2020). It has been shown to be a valid, reproducible scale that identifies patients who are ready to return to their preinjury sport. It is based on the same model as the ACL-RSI and Shoulder Instability RSI (Webster et al., 2008; Gerometta et al., 2018). The ALR-RSI is a one dimensional 12 item scale that measure 3 types of responses believed to be associated with the RTS following sports injuries (Podlog et al., 2014): emotions (five items), confidence in performance (five items), and risk appraisal (two items) (Figure 7). Each item is rated from 0 to 10 and the total score is determined by adding the values of the 12 answers then dividing the result by 1.2 to obtain a percentage. High scores correspond to a positive psychological response. A highly significant difference was found between the subgroup of patients who successfully returned to sport and those who did not 3 years after ankle ligament reconstruction (68.8 vs. 45.0 respectively) (Sigonney et al., 2020). This scale is used increasingly frequently after surgery and was recently validated among individuals with CAI who underwent a modified Broström-Gould procedure (Pioger et al., 2022). Results showed higher scores (61.9) in patients who returned to sports compared to those who no longer practiced a sport (43.4) 2 years after repair. This score has also been recently translated and validated in French (Ajaka et al., 2022).

## Discussion

There are no existing objective RTS criteria after lateral ankle sprain, chronic ankle instability or surgical stabilization. Two recent systematic reviews (Tassignon et al., 2019; Wikstrom et al., 2020) have been published suggesting that it is important to include functional tests associated with PROM in RTS decision-making, thus we performed a narrative review to provide clinicians with concrete outcomes in the RTS process. Based on the definition of copers we determined simple, accessible, and reliable criteria to manage RTS in patients with CAI, LAS or after surgery.

Numerous other variables and tests could be considered for RTS decision-making in CAI besides those included in this narrative review. Our tests and questionnaires were chosen based on the best available scientific evidence on functional tests and questionnaires in patients with CAI to support clinicians in the complex RTS decision-making process. These tests and questionnaires should be used sensibly when determining the RTS. The scores must be interpreted in relation to the individual patient, and in relation to other tests and potential variables of interest. Moreover, the results should not be interpreted separately. A single test score or questionnaire is not enough to make a RTS decision. In addition, the purpose of rehabilitation is not to train athletes to pass predetermined criteria without being ready for the RTS, but to assess whether athletes are truly ready to safely RTS. In other words: train the athlete, not the test. It is worth mentioning that this review focused on functional tests and self-reported questionnaires assessing patient function and apprehension after lateral ankle sprain or ankle instability (operated or not). Other components of the PAASS have been previously described and assessed in the 2019 consensus statement of the International Ankle Consortium about clinical assessment of acute lateral ankle sprain injuries (ROAST), while certain other tests require modalities or equipment that is not always available in daily practice (Delahunt et al., 2018; Smith et al., 2021).

Because hopping and balancing tests assess different components of ankle function (strength, power, agility, proprioception and neuromuscular control) it seems relevant to combine them for RTS decision-making in patients with CAI. Ko et al. (2017) suggest combining functional performance tests rather than a single test to improve the clinical value of testing. Specifically, a combination of the SHT and SEBT was found to have the greatest clinical value (Rosen et al., 2019). Since the meta-analysis by Houston et al. (2015) did not show any difference in ankle function between copers and healthy individuals, it seems reasonable for clinicians to target the coper FAAM_adl_, FAAM_sport_ and ALR-RSI cut-off values. Like Smith et al. for PAASS, we did not include certain tests described in ROAST because by the time the RTS decision is made, these items should have already been acquired. Nevertheless, we chose to integrate the FAAM with an increased threshold (95%), as recommended by Hertel and Corbett (2019). Clinicians who wish to further validate the RTS decision can also consider ankle muscle strength tests (Terrier et al., 2017), proprioception assessments such as joint position recognition testing (McKeon and McKeon, 2012), neurocognitive functional performance (Tassignon et al., 2020) and sport-specific performance tests (Clanton et al., 2012).

### Ankle Muscle Strength Testing

The assessment of ankle evertor muscle strength also appears to be a key parameter to manage RTS after acute ankle sprain as well as CAI (Hertel and Corbett, 2019; Smith et al., 2021). More precisely, eccentric ankle evertor performance is highly important because it takes part in the active control of sudden ankle inversion (Munn et al., 2003; Collado et al., 2009; Terrier et al., 2014). However, this deficit has rarely been evaluated in clinical practice (Amaral De Noronha and Borges, 2004; Plante and Wikstrom, 2013). Thus, an isokinetic evaluation is still considered to be the gold standard procedure for research, although this methodology cannot be easily used in daily practice due to costs, space, bulk and time which are barriers for clinicians. Moreover, because subjects are sitting (i.e., not in a weight-bearing position) during the test, torque data must be normalized to body mass for comparison purposes. Alternative testing methods such as hand-held dynamometers have been shown to be reliable and more practical for clinicians (Spink et al., 2010). However, it should be remembered that open kinetic chain ankle isokinetic testing and hand-held dynamometers cannot match the closed kinetic chain function of ankle evertors (Dvir, 2003; van Cingel et al., 2009; Edouard et al., 2011). Several years ago our team (Terrier et al., 2014, 2017; Hertel and Corbett, 2019) proposed an easy, accessible test for this purpose. We showed that the ability to control weight-bearing ankle inversion was altered in CAI compared to healthy individuals. In particular the peak angular velocity was significantly higher among individuals with CAI during a controlled unipodal weight-bearing inversion task. Thus, neuromuscular control in a situation requiring strength can be assessed with a simple test using a simple (angular speed) measurement (Terrier et al., 2021). Although our proposal used the specific device (Myolux™) we feel that the ability to control ankle weight-bearing inversion could be easily and rapidly obtained without specific equipment.

### Neurocognitive Functional Testing

Current functional performance tests used for RTS decision-making assess certain aspects of physical performance and quality of movement in a closed environment (Hegedus et al., 2015, 2016; Chimera and Warren, 2016). Thus, these tests only include pre-planned motor tasks and thus ignore essential neurocognitive features of sports such as adaptability, decision-making, uncertainty, responding to stimuli, etc. Neurocognitive functional tests could also have added value because lower neurocognitive performance as well as the addition of cognitive load to physical performance have been associated with an increased risk of sports injuries (Swanik et al., 2007; Brown et al., 2009; Wilkerson, 2012; Herman and Barth, 2016; Seymore et al., 2017).

**Figure 8:**
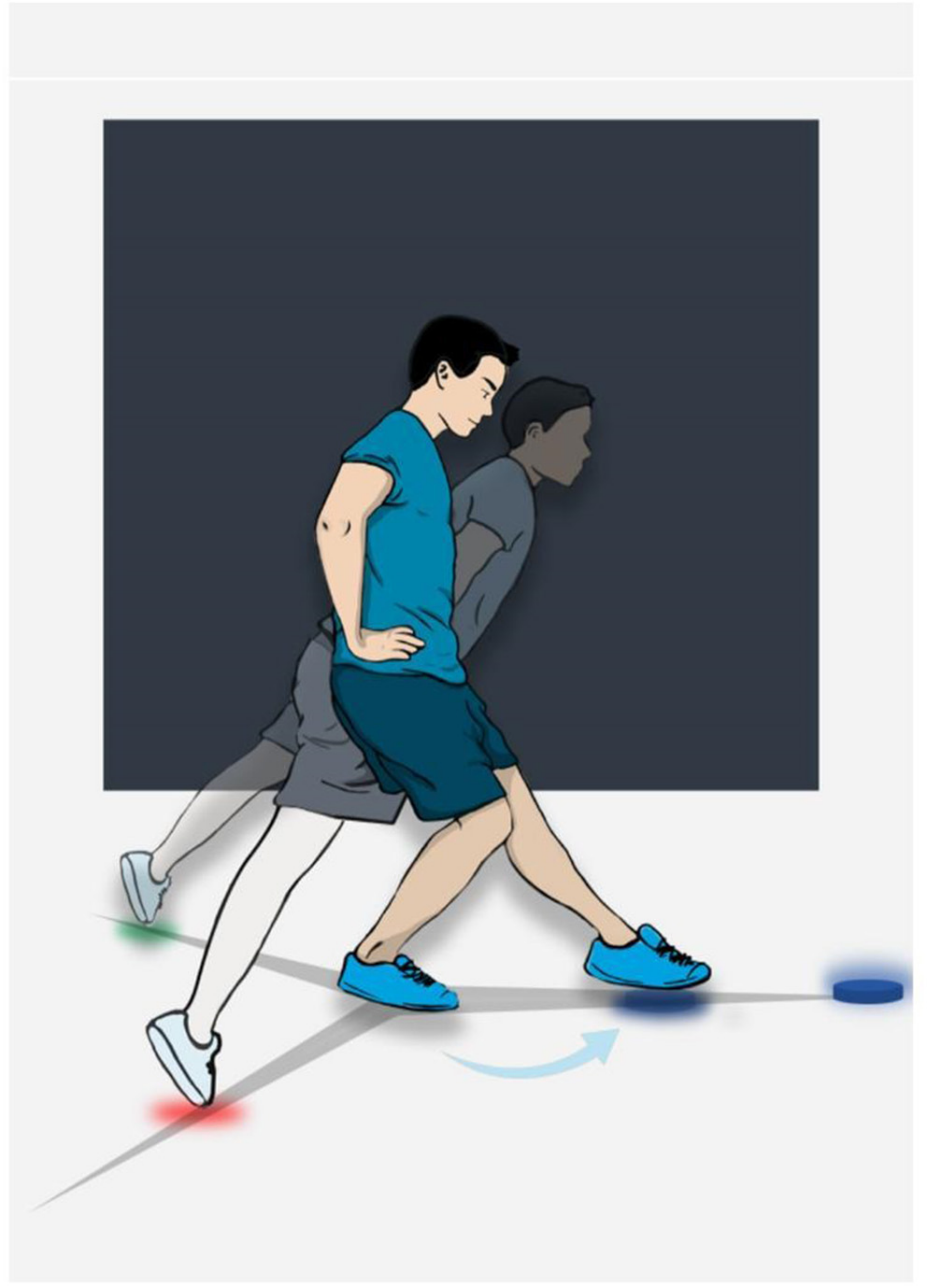
Example of neurocognitive testing (reactive balance test). In this figure, the color blue is shown and the participant then has to decide which of the three LED-lights (red, green or blue) need to be extinguished. The participant correctly turns off the blue light on the anterior axis in this case.

Examples of neurocognitive functional tests include the reactive balance test (Verschueren et al., 2019; Tassignon et al., 2020) and neurocognitive hop tests (Millikan et al., 2019; Simon et al., 2020). These neurocognitive functional tests possess good to excellent reliability. The reactive balance test uses the Y-balance test in combination with a neurocognitive task, so that participants must extinguish the correct LED-light as fast as possible while maintaining single-leg balance (Figure 8). The neurocognitive hop test challenges the participant in different ways by slightly changing the instruction and set-up when performing these tests. One version of a neurocognitive hop test makes participants wait for the “go” signal before they react as quickly as possible to the stimulus while also jumping as far as possible. Another version lets participants perform the same task but only when the correct color is shown. Even though research on these neurocognitive functional tests is in its infancy, they could provide innovative assessment of patients with CAI during rehabilitation and when making RTS decisions.

### Applicability in the Return to Sport Continuum

One of the specificities of the management of ankle sprains and chronic instability is that many patients keep performing their usual activities despite their pathological status. Furthermore, most of patients do not seek appropriate treatment. However, in the acute phase of injuries, the disability or pathomechanical impairments lead the patient to consult a health care professional and relative rest (PEACE & LOVE) is therefore recommended (Dubois and Esculier, 2020). In order to limit the risk of recurrence or episodes of giving-way during the treatment, it is the clinician's responsibility to discuss with the patient to consider discontinuing at risk activities temporarily, and to explain possible implications of continuing the at-risk activities at the moment. In general, any sport involving cutting maneuvers or landing should be avoided. It is preferable to perform tasks and activities with a lower impact such as straight line running. In this phase, it is necessary to follow the recommendations indicated in the RTP continuum (Ardern et al., 2016; Tassignon et al., 2019; Smith et al., 2021). Communication and trust between clinician and patient is vital during rehabilitation to optimally prepare the patient to RTS. Therefore, the patient's voice should be heard when making this decision (shared decision-making). Taking these measures would give the clinician the opportunity to provide comprehensive rehabilitation contents without the risk of making the injury worse and avoid recurrences. The test battery and questionnaires proposed in this paper can then be implemented to validate the transition from return to participation (Stage 1) to the return to sport (Stage 2).

For example, in the case of a basketball player, return to run (Stage 1) may be allowed following the validation of the ROAST (Delahunt et al., 2018). However, the return to full basketball training (Stage 2) requires maneuvers involving, a.o. numerous changes of direction. These specific movements cause significant stress on the ankle in the frontal plane, which requires skills that were not assessed in the ROAST and justifies the validation of the tests and questionnaires that we propose.

On the other hand, the transition to the third stage of the RTS continuum (return to performance) requires the validation of more sport-specific tests investigating if the player has returned to his or her pre-injury level of play.

### Sport-Specific Testing

Depending on the time and resources of the clinician and athlete, sport-specific tests can be added to the functional tests and questionnaires. The primary purpose of sport-specific tests is to measure the patient's actual performance level and provide sport-specific training goals. Furthermore, quantitative and qualitative impairments can also be observed during sport-specific tests and used to guide (re-)injury prevention and rehabilitation strategies. For example, the T-Agility Test and the Illinois Test are commonly described to assess agility and the ability to perform cutting maneuvers in athletes and could be used as RTP criteria (Clanton et al., 2012; Hachana et al., 2013). The experts who were consulted in the development of the PAASS framework suggested including sport-specific functions for RTS decision-making in patients with a lateral ankle sprain injury (Smith et al., 2021). Based on this, sport-specific tests should also be considered for RTS decision-making in CAI populations. Sport requirement analyses are recommended to select the most relevant sport-specific tests. These requirement analyses can be divided into four large sport-specific categories: exercise physiology, biomechanics, muscle-tendon functioning, and essential skills. They can be used together to create a unique profile for each sport. For instance, the requirements of a volleyball player will be different in all four categories than those of a handball player, with certain comparable requirements. However, a detailed discussion of adding sport-specific tests to the functional tests and questionnaires to decide on the RTS in patients with CAI is beyond the scope of this article.

It is also worth mentioning that test performance may depend on the type of sport. Stiffler et al. (2015) showed that scores on the SEBT varied according to gender and type of sport. Thus, the performance on different tests in relation to the potential risk of recurrence needs to be interpreted with caution taking into consideration the athlete's sport. In addition, comparisons between sports are difficult. Practitioners working with athletes need baseline assessments to use as target criteria for the RTS. Comparisons with the healthy ankle (Limb Symmetry Index) can also help in the RTS decision.

Finally, the evaluation of the sensation of ankle instability during tests and sports tasks should be taken in account. Caffrey et al. (2009) highlighted the importance of reporting instability during the Figure-of-8 hop test and SHT as they could help identify patients with severe functional ankle instability.

## Conclusion

No objective return to sport criteria exist after lateral ankle sprain. This narrative review and expert opinion provide values for several relevant functional tests and self-reported questionnaires that target ankle impairment to help clinicians in return to sport decision-making. The single leg stance test on a firm surface, the modified version of the star excursion balance test, the single hop test and the figure-of-8 test appear to be the most clinically relevant functional tests for individuals with lateral ankle sprain, chronic ankle instability or patients after surgery. Moreover, the Foot and Ankle Ability Measure combined with the Ankle Ligament Reconstruction-Return to Sport after Injury questionnaires seem to be the most relevant scores for the functional assessment of these patients. Further studies are needed to determine the validity of this cluster to discriminate individuals who can successfully return to sport at the preinjury level.

## Author Contributions

BP, AH, RL, and FF contributed to the conception and design of the study. BP, RT, and FF performed the literature search. All authors were involved in the key words description, reviewing process, and participated in the expert opinion. All investigators wrote specific sections of the manuscript. All authors contributed to manuscript revision, read, and approved the submitted version.

## Funding

This work was funded by Chirurgie du Sport, Paris.

## Conflict of Interest

The authors declare that the research was conducted in the absence of any commercial or financial relationships that could be construed as a potential conflict of interest.

## Publisher's Note

All claims expressed in this article are solely those of the authors and do not necessarily represent those of their affiliated organizations, or those of the publisher, the editors and the reviewers. Any product that may be evaluated in this article, or claim that may be made by its manufacturer, is not guaranteed or endorsed by the publisher.
